# Health technology assessment (HTA) of optoelectronic biosensors for oncology by analytic hierarchy process (AHP) and Likert scale

**DOI:** 10.1186/s12874-019-0775-z

**Published:** 2019-07-05

**Authors:** Giovanni Improta, Antonietta Perrone, Mario Alessandro Russo, Maria Triassi

**Affiliations:** 1 Department of Public Health, University of Naples “Federico II”, Naples, Italy; 2 Service of Clinical Engineering, Health Technology and HTA - University Hospital AOU Federico II of Naples, Naples, Italy

**Keywords:** Health technology assessment, AHP methodology, Multicriteria decision problems, Likert scale

## Abstract

**Background:**

The multicriteria decision method (MCDM) aims to find conflicts among alternatives by comparing and evaluating them according to various criteria to reach the best compromise solution. The evaluation of a new health technology is extremely important in the health sciences field. The aim of this work is to evaluate a new health technology to assay thyroglobulin in patients with differentiated thyroid cancer to improve its service from an organizational point of view, by planning new and appropriate training activities, ensuring proper use of resources and satisfying the needs of different users.

**Methods:**

The evaluation was performed using two methodologies: the analytic hierarchy process (AHP) and the Likert scale. The AHP is a multicriteria decision approach that assigns a weight to each evaluation criterion according to the decision maker’s pairwise comparisons of the criteria. The Likert scale is a psychometric scale employed to study the degree of user satisfaction by measuring opinions.

**Results:**

Results show the need of particularly improving clinical efficiency, effectiveness, and return on sales (ROS) related to the technology; technological safety, human resources and other parameters do not need to be improved because of the high satisfaction results of the users.

**Conclusions:**

The application of both methods provided the necessary information to improve the quality of the service, allowing the decision maker to identify the most valuable service features and to improve these to ensure user satisfaction and to identify possible service improvements.

## Background

The analysis of the multicriteria decision method (MCDM) aims to identify conflicts among alternatives by comparing and evaluating them according to various criteria to reach the best compromise solution [1–4]. Today, many MCDM methods are in use [5–7]. Among these, the Analytic Hierarchy Process (AHP) is of considerable interest. The AHP, or hierarchical analysis, is a methodology, developed in the 1970s by Thomas L. Saaty [8], which evaluates a set of alternatives and creates a final version of the same problem by splitting the problem into many sub-problems for decision making [1, 9–20].

This method can handle a large number of different factors, which are often in conflict with each other, and it can also compare different alternatives in relation to a number of criteria [21–29].

Among the several application of AHP, this approach has been applied by Suner et al. [30, 31] to the management of rectal cancer. They constructed a sequential decision tree for the best treatment decision process, using priorities determined by the AHP method. Moreover, they were able to develop a web-based clinical decision support tool for physicians in the selection of potentially beneficial treatment options for patients with rectal cancer by combining the AHP, which determines the priority of criteria, and decision tree that formed using these priorities. More recently, Suner et al. [32] also applied AHP in the evaluation of infectious diseases to determine the best hand hygiene preference of the infectious diseases and clinical microbiology specialists to prevent transmission of microorganisms from one patient to another. They examined opinions of the specialists with two widely used multi-criteria decision analysis methods, the Multi-Attribute Utility Theory (MAUT) and the AHP, showing that both decision models indicate that rubbing the hands with alcohol-based antiseptic solution is the most favorable choice for specialists to prevent nosocomial infection.

Through the AHP methodology, a weight is assigned to each alternative, relying on the reviews provided by the purely qualitative decision maker, and each weight vector is placed in a final matrix that is used to sort the priority of each alternative. Several works discuss the use of AHP in solving health technology assessment problems [33, 34]. These studies demonstrate the ability of AHP to facilitate the understanding of the criteria and the priorities to successfully evaluate hospital technologies. Therefore, the AHP may be considered as a decision support tool for the Health Technology Assessment (HTA) projects [35–38].

This work focuses on a specific clinical application of the HTA. Indeed, HTA enables the analysis and assessment of health technologies, considering all medical-clinical, organizational, economic, social, legal and ethical implications, both directly and indirectly caused, and both short- and long-term implications [39, 40].

Many scientific papers [41, 42] focus on the innovative contribution of HTA [43–46] using the project management methodology to improve the quality of health services, in particular aiding the optimal allocation of biomedical systems, by evaluating all safety, ethical, legal and social, economic, technical and technological, and organizational parameters. If these requirements are met, hospitals can proceed with installing the system in the assessed area only; otherwise, the target area will not comply with the installation of the system, and then the assessment can be repeated for another area or to choose a different type of system.

In this work, the AHP approach is applied to a HTA [36, 43, 46–49] issue to evaluate an assessment that involves the use of optoelectronic systems in oncology (specifically, cancer of the thyroid). In addition, it offers the most suitable solution for its application since the choice of medical technology produces complex interactions among the variables involved [50]. Customer satisfaction is measured and evaluated not only by the AHP [51–55] but also using the Likert scale. The Likert scale is used to evaluate the degree of user satisfaction by measuring their opinions. This enables numerically quantifying the satisfaction of users in relation to the defined items, thereby allowing us to investigate the experts’ preferences about various defined “items”.

## Methods

The methodological approach adopted to evaluate the perceived satisfaction is the questionnaire, which was administered to a sample of 80 users. Two different methods were implemented to analyse the data acquired by the questionnaire, the Likert scale and the AHP, which have been widely used in the literature in many application areas and in the health care sector [56–58].

The Likert scale is a psychometric scale widely used in many different research areas to evaluate questionnaires. Questionnaires assessed by the Likert scale formula usually a n-point scale (generally, from 4- to 7-point scale). Respondents are forced to choose in even-numbered scales because odd-numbered scales allow for indecision or neutrality. Questions designed using the Likert scale must either be in agreement or disagreement. This method has been found to be reliable at obtaining valid measurements of training effectiveness, reaction (satisfaction with conducted training), and overall training impact at work [59]. The Likert scale also has some drawbacks, including limitations due to the honesty of respondents in answering the questions, reproducibility and validity of the results [60].

AHP is a multicriteria decision making tool for organizing and analysing complex decisions based on mathematics and psychology. AHP incorporates group census from a questionnaire by comparing each element and geometric mean to arrive at a decisive solution.

AHP is used around the world in a wide variety of decision situations, in fields such as government, business, industry, healthcare and education, to plan, select best alternatives, allocate resources, resolve conflicts and optimize decisions [61]. Training programmes are efficiently evaluated by the AHP as this method provides the highest value and ranks the content and trainer based on logistics [62].

**Fig. 1:**
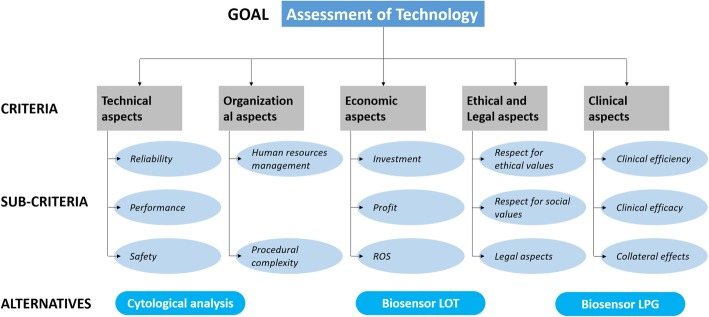
Top-level criteria and indicators of each criterion

The top-level criteria and indicators of each criterion highlighted by HTA are listed in Fig. 1.

This approach provides a hierarchical decomposition of the problem, first into goals, criteria, sub-criteria and alternatives, and then calculates the global sub-criterion weight.

It is important to consider all aspects related to the definition of the minimum technical quality of an evaluated technology, as these aspects can subsequently result in the minimum level guarantee of the effectiveness and efficiency of the process under study.

To assess the technical aspects, the following indicators/sub-criteria are considered:

- Technological efficiency is a technological performance level indicator that measures the amount of useful work produced, depending on time and available resources. It represents the ability to act with maximum efficiency and minimal waste of resources. The sum, at each time step of the executed exams, is the indicator of the sub-criterion technological efficiency; the higher this indicator, the greater the efficiency of the technology:
  1 $$ {\sum}_{timestep} executed\ exams\kern0.5em $$
- Reliability aims to describe and measure the operation of the devices or equipment. For any given system, this measure quantifies the degree of confidence we have in the good functioning of the system, i.e., the fulfilment of the purposes for which the system itself is designed and built. An important indicator of the reliability of the technology, especially for a device, is the number of faults that the technology undergoes per unit of time. The sum at each timestep of queued examinations for faults is the indicator of higher reliability; the higher this indicator sub-criterion, the higher the number of tests for faults, so the technology is less reliable:
  2 $$ {\sum}_{timestep} queued\ exams\ for\ faults $$
- Safety technology of the equipment depends on strict compliance with the requirements of the equipment, in terms of both design and construction, during installation, use and maintenance over time. To assess this indicator, another system, separate from the one above, is constructed. The indicator includes a number of variables and constants: visual evaluation, compliance with safety standards, the availability of user manuals, the presence of alarm signals and control systems and the status of the use of the rooms and facilities. The sum of the values of these variables (each of which will have a value from 1 to 10) constitutes the indicator of the sub-criterion “Security Technology”. The higher the level of security associated with the technology, the greater the indicator. By normalizing these three vectors at each time step, the local weight of the alternatives relative to the three indicators is obtained.

The following indicators/sub-criteria are considered in the assessment of the organizational aspect:

- the procedural complexity is expressed as:
  3 $$ procedural\ Complexity=\frac{total\ exams\ average\ executed\ daily}{total\ exams\ to\  be\  performed\ daily} $$

This relationship, with appropriate adjustments (which can vary from days to hours), is the indicator of the procedural complexity, and this sub-criterion indicates the hours spent to run the exams for each alternative. The greater the number of hours, the greater the procedural complexity associated with the technology.

- the human resources.

The number of staff needed and available to run sampling and analysis is the indicator of human resources, and the greater the sub-criterion of this indicator, the greater the necessary staff for the technology:

4 $$ human\ resources= medical\ specialists+ biologists $$

By normalizing these two vectors at each timestep, the local weight of the alternatives for the two indicators is obtained.

The following indicators/sub-criteria are considered in assessing the economic aspects:

- Investments are considered mainly for the purchase cost of the technology. The relationship between investment and the purchase cost of the technology is the indicator of the sub-criterion investments. The higher this indicator, the smaller the investment associated with the technology:
  5 $$ investiments=\frac{investiment}{purchase\ cost\ of\ the\ system} $$
- The profit is the profit or loss from business management over a period of time. The sum of the net profits at each timestep is a sub-criterion for the usefulness indicator: the higher this indicator, the greater the gains associated with the technology:
  6 $$ profit={\sum}_{timestep} net profit $$
- The ROS, or return on sales, measures the sales profitability and lucrative revenue streams typical of the capacity of the enterprise. The ratio of net operating margin (MON) and net revenues, obtained by multiplying the tests processed with drug revenues, constitute the sub-criterion for ROS. The higher this indicator, the more profitable the product (in this case medical examination):
  7 $$ ROS=\frac{MON}{net\  revenues} $$

By normalizing these three vectors at each time step, the local weight of the alternatives are obtained for the three indicators.

We consider any provision that can enclose two primary qualities, such as effectiveness and efficiency, as appropriate, and at the same time, we consider both acceptable by those who receive and dispense them [63].

Therefore, to assess the clinical aspects, the following indicators/sub are considered:

- Clinical efficiency [64]. The difference between the sum of the examinations that require no repetitions (between sampling and analysis) and the sum of examinations requiring repetitions (between sampling and analysis) at each timestep constitutes the sub-criterion: the greater the clinical efficiency indicator, the greater the efficiency of the technology:
  8 $$ Clinical\ efficiency=\Big[\left(\sum tests\ that\ require\  no\  additional\ withdrawals\right)+\left(\sum tests\ that\  do\  no t\ require\ further\ investigation\right)-\left(\sum exams\ that\ require\ further\ withdrawals\right) $$
- Effectiveness. The ratio of the sum of effective tests and the sum of incoming requests is the sub-criterion for the effectiveness indicator: the greater this efficiency indicator, the greater the degree of effectiveness of the technology:
  9 $$ \mathrm{Effectiveness}=\frac{\sum_{\mathrm{timestep}}\mathrm{effective}\ \mathrm{tests}\ }{\sum_{\mathrm{timestep}}\mathrm{incoming}\ \mathrm{requests}} $$
- Side effects. The sum of the examinations that have side effects and effective examinations with error constitutes the side effects sub-criterion: the greater the side effects indicator, the more side effects the technology has:
  10 $$ Side\ effects=\sum \limits_{timestep} examinations\ that\ have\ side\ effects+\sum \limits_{timestep} effective\ examinations\ with\ error $$

The local weight of the alternatives relative to these three indicators is obtained by normalizing these three vectors at each timestep.

The following indicators/sub-criteria are considered for the purposes of social, ethical and legal aspects.

- the **respect for social aspects** refers to following the principle of justice, which provides for the equality of treatments based on clinical conditions, i.e., the possibility that the technology at issue has equal access for all patients, especially in terms of the costs it entails. The sum of examinations is the indicator of the sub-criterion of compliance with social aspects; the higher this indicator, the more the testing, and hence the lower economic unavailability due to missed examinations. This means that the technology is more respectful of social aspects.
  11 $$ \mathrm{R} espect\ for\ social\ aspects={\sum}_{timestep} Examinations $$
- The **respect for ethical principles** refers to the moral significance of biomedical and biotechnological practices at the borders of right and not right concerning who you can hire, the rights of the person, the decisions entitled to the people, the decision criteria and values at stake. Additionally, this criterion includes a sense of current concepts such as respect for human life and respect for the dignity of the person. The sum of examinations not performed for ethical reasons, related to both the surgeon and the patient, is the indicator of the sub-criterion for compliance with the ethical principles:
  12 $$ \mathrm{Respect}\ \mathrm{for}\ \mathrm{ethical}\ \mathrm{principles}={\sum}_{timestep}\mathrm{examinations}\ \mathrm{not}\ \mathrm{performed}\ \mathrm{for}\ \mathrm{ethical}\ \mathrm{reasons}\ \mathrm{related}\ \mathrm{to}\ \mathrm{the}\ \mathrm{surgeon}+{\sum}_{timestep}\mathrm{examinations}\ \mathrm{not}\ \mathrm{performed}\ \mathrm{for}\ \mathrm{ethical}\ \mathrm{reasons}\ \mathrm{related}\ \mathrm{to}\ \mathrm{the}\ \mathrm{patient} $$
- The **respect for legal issues** implies that the field of medical equipment and technology must be regulated through legislation, the so-called European directives, technical standards issued by the relevant standardization bodies. To calculate this indicator, variables were given a score between 1 and 10, where 1 is the best value, and 10 the worst. The indicator was calculated by adding up all the scores. The smaller the indicator, the higher respect for the legal issues. The sum of these variable provides the respect for legal issues indicator:

Thus,

legal = noise + electromagnetic pollution + respect the value of life +.

health risk + compliance with mandatory standards + lawsuits.

By standardizing the three vectors obtained at each timestep, the local weight of the alternatives is obtained and compared for the three indicators.

### Data processing using the AHP (analytic hierarchy process) and confirming the consistency of the matrices

The scores from the comparisons in the AHP are used to construct a matrix of pairwise comparisons. To aggregate the individual judgements, the weighted geometric mean method (WGMM) is used according to the following formula:

13 $$ \prod \limits_{k=1}^N{\left({a}_k\right)}^{\beta_k} $$

by applying the constant weight 1/N, the equation is rewritten:

14 $$ \prod \limits_{k=1}^N{\left({a}_k\right)}^{\raisebox{1ex}{$1$}\!\left/ \!\raisebox{-1ex}{$N$}\right.}=\sqrt[N]{\prod \limits_{k=1}^N{a}_k} $$

and by applying a logarithm operation, the computation is simplified as a sum:

15 $$ {10}^{\frac{1}{N}\sum \limits_{\mathrm{k}=1}^N\log {a}_k} $$

Obviously, as the geometric mean returns a non-integer value, we round the obtained value to the nearest integer.

From the average values thus calculated and the properties of the matrix of pairwise comparisons, a matrix inherent to the three main categories can be constructed. A matrix was also constructed for all other pairwise comparisons (matrices are reported in Table 3).

Now we need to analyse the consistency, which is accomplished by calculating the ratio of texture CR = (CI/RI) where:

16 $$ \mathrm{CI}=\frac{\uplambda_{\mathrm{max}}-\mathrm{n}}{\mathrm{n}-1} $$

and RI is a random index that assumes a certain value depending on the order of the array. *λ*_*max*_ is the largest eigenvalue, *n* is the matrix dimension, and *RI* is the Saaty random consistency index.

According to Saaty (2008) [65], if the consistency ratio exceeds 0.1, the set of judgements may be too inconsistent to be reliable. In practice, CRs of more than 0.1 sometimes are accepted. If CR equals 0, then the judgements are perfectly consistent [66].

All data elaboration and analysis fo the AHP has been carried out by using Matlab (Mathwork).

## Case study and implementation of the AHP

**Fig. 2:**
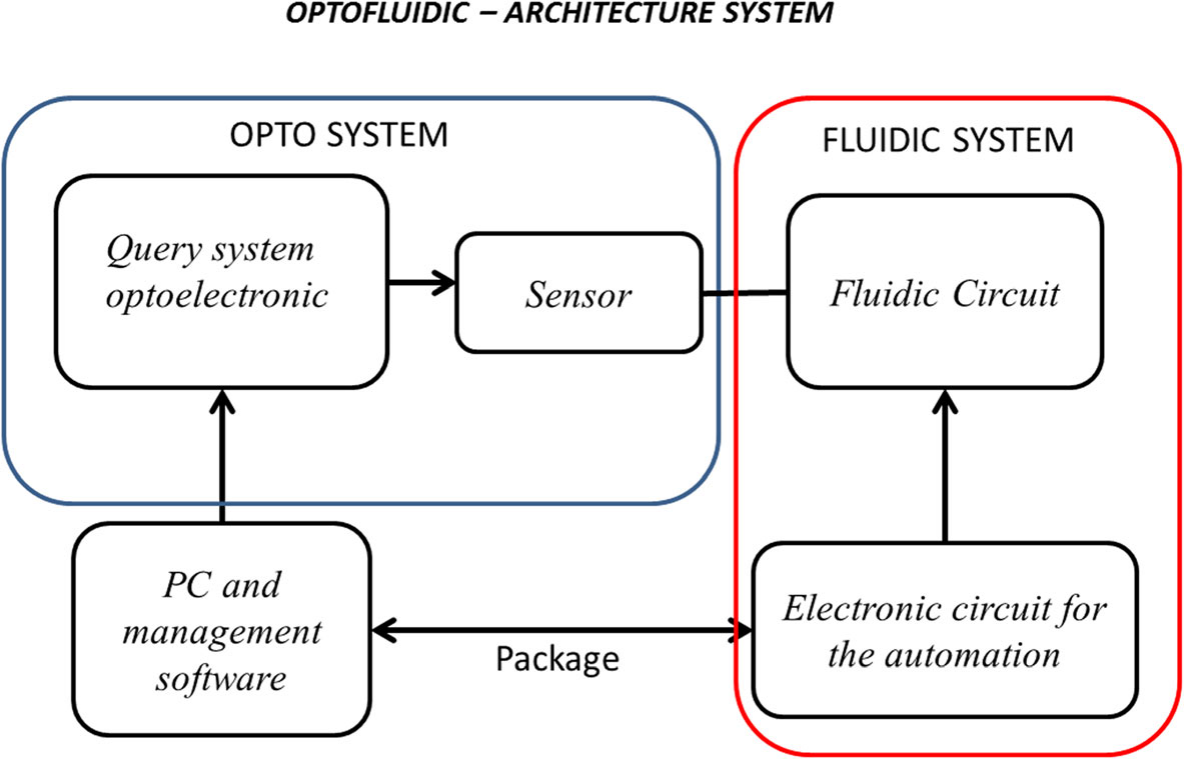
Representation of optofluidic system. Architecture of the system

The present case study assessed two new health technologies used in oncology (opto system and fluidic system), as displayed in Fig. 2. These two systems combine fibre optics, electronics and fluid dynamics to determine the levels of thyroglobulin (Tg) in needle-aspiration samples taken from the thyroid. Several treatment options are available for the management of thyroid cancer. Tg is a large glycoprotein composed of two identical polypeptide chains that form a molecule of approximately 660 kDa and localized in the follicular thyroid colloid. Currently, Tg is assayed in suspicious lymph aspirates, the results of which are then used in a cytological analysis.

**Fig. 3:**
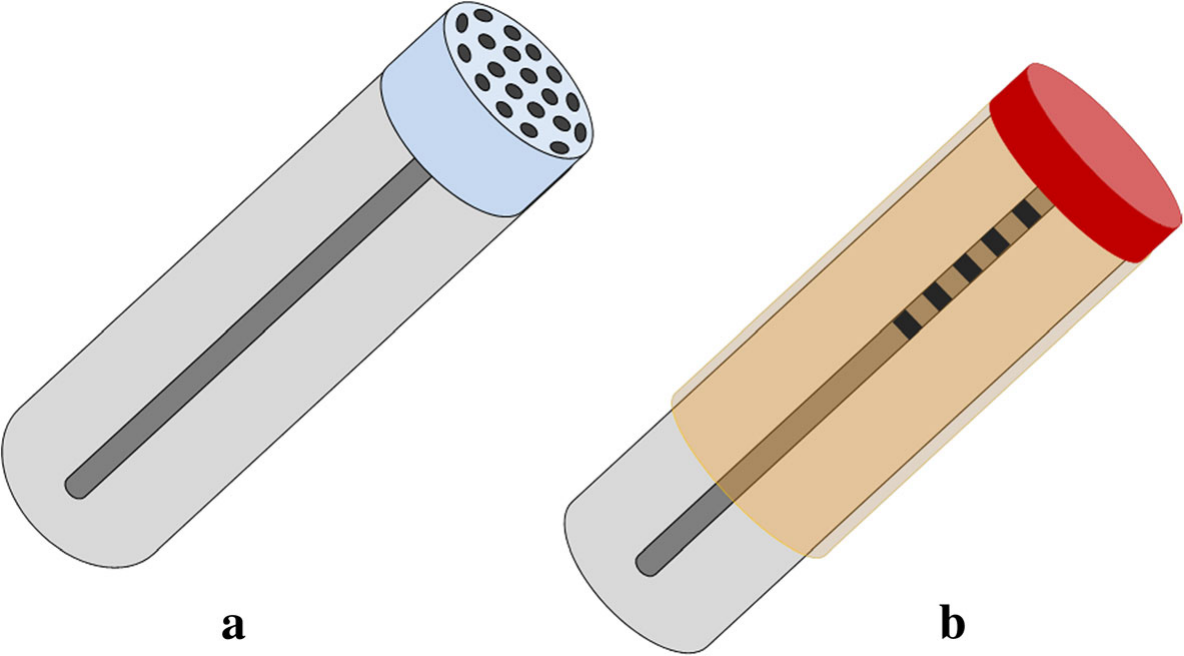
Schematic representation of the two different types of sensors: with integration and patterning on the tip of the fibre, LOF (**a**), and based on the long-wheelbase patterns coated with nanoscale layers of functional polymer materials, LPG (**b**)

To specify the concentration of Tg (with a sensitivity limit above 100 ng/ml), biosensors with fibre-optic transducers should be characterized by:

- high sensitivity, to reveal even small concentrations of tumour markers in biological liquids;
- high selectivity, to prevent the influence of other substances in the lymph node that are irrelevant to the analysis of sick nodules;

Two different types of fibre-optic transducers are considered to meet the sensitivity requirement:

1. the first transducer, named a lab on fibre, (LOF), integrates nanoscale metallic patterning on the tip of optical fibres. The resulting structures are plasmonic crystals capable of trapping light at a specific wavelength of resonance (Fig. 3);
2. the second configuration, called LPG (long period gratings), involves the use of long-wheelbase patterns inscribed within the core of the fibre, which is covered with nanoscale layers of functional polymeric materials (Fig. 3 b).

However, the surface must be appropriately engineered to meet the selectivity, so that only the target molecule (and not other molecules present in the biological solution) is immobilized and detected by the sensor.

An optofluidic system integrates fibre-optic sensors with fluidic circuits that can manage the flow of biological solutions while ensuring the correct operation of the sensor. It consists of two major subsystems:

- the “*opto”* subsystem is responsible for analysing biological fluids sampled by needle aspiration and indicating the concentration of tumour markers contained therein;
- the “*fluidic”* subsystem manages the sampled body fluids and ensures proper performance of the analysis.

Both are managed by the same software with a user-friendly graphical interface that can be used even by unskilled personnel.

## Results and discussion

### Data processing using AHP (analytic hierarchy process)

The judgements used to construct the matrices of pairwise comparisons for the AHP were provided in collaboration with the National Research Council staff working in the field of biosensing systems.

Three options are considered, including decisions for each criterion. The first option is cytological analysis, which represents AS IS, i.e., the current method of assessing Tg levels. The other two options are the two biosensors that should be used to determine Tg, the one with the sensor on the tip of a fibre (LOF) and the one with the fibre along the surface (LPG), which represent TO BE. The elements of the array represent the relative importance of one criterion over another, using the scale proposed by Saaty and reported in Table 1*.*

**Table 1:** Saaty’s pairwise comparison scale

Intensity of importance	Degree of preference	Description
1	Equal importance	Two activities contribute equally to the objective.
2	Weak	
3	Moderate importance	Experience and judgment slightly to moderately favour one activity over another.
4	Moderate plus	
5	Strong importance	Experience and judgment strongly or essentially favour one activity over another.
6	Strong plus	
7	Very strong or demonstrated importance	An activity is strongly favoured over another and its dominance is showed in practice.
8	Very, very strong	
9	Extreme importance	The evidence of favouring one activity over another is of the highest degree possible of an affirmation.
Reciprocals of above values	If activity i has one of the above non-zero number assigned to it when compared to with activity j, then j has the reciprocal value when compared with i	Reasonable assumption.
1.1–1.9	If the activities are very close	May be difficult to assign the best value but when compared with other contrasting activities, the size of the small numbers would not be too noticeable, yet they can still indicate the relative importance of the activities.

On the basis of the Saaty’s scale, the following Matrix of pairwise comparisons has been built (Table 2). Each value reported in the Table 2 represents the geometric logarithmic mean of the degree of preference of the 80 users according to the Saaty’s fundamental scale.

**Table 2:** Matrix of pairwise comparisons with respect to policy goals

Assessment of healthcare technology	Technical aspects	Organizational aspects	Economic aspects	Clinical aspects	Social, ethical and legal aspects
Technical aspects	1	1/3	1/7	1/5	1/5
Organizational aspects	3	1	1/5	1/3	1/3
Economic aspects	7	5	1	3	5
Clinical aspects	5	3	1/3	1	3
Social, ethical, legal aspects	5	3	1/5	1/3	1

The matrix in Table 2 is positive, mutual and consists of finite elements. For a matrix of 5 × 5 order, such as that in Table 2*, RI = 1.12*. The maximum eigenvalue associated with the matrix is calculated:

17 $$ {\uplambda}_{\mathrm{max}}=5.3185\ \mathrm{n}=5 $$

18 $$ C\mathrm{I}=\frac{5.3185-5}{4}=0.0796 $$

19 $$ C\mathrm{R}=\frac{0.0796}{1.12}=0.0711=7.11\%<10\% $$

Therefore, the matrix can be considered consistent.

The eigenvector associated with the maximum eigenvalue can now be calculated:

20 $$ {\mathrm{w}}_1=\left[0.0703;0.1403;0.8582;0.4194;0.2509\right] $$

The normalized vector is:

21 $$ {\mathrm{w}}_{1\mathrm{N}}=\left[0.0404;0.0807;0.4935;0.2412;0.1443\right] $$

which is the local weight vector with respect to the policy goals.

Finally, by aggregating all the global weights for each of the three alternatives, we obtain the final sorting vector, which indicates the priorities of the alternatives, i.e., the result of the decision problem.

22 $$ \mathrm{Cytological}\ \mathrm{Analysis}:0.0114+0.00042+0.00960+0.0105+0.00512+0.0228+0.0270+0.0080+0.0114++0.0258+0.01632+0.0077+0.00954+0.1774=0.334414; $$

23 $$ \mathrm{Biosensor}\ \mathrm{LOF}:0.00320+0.00320+0.00162+0.0288+0.00149+0.0256+0.0961+0.0961+0.0450+0.0046+0.00544+0.0018+0.0477=0.36065; $$

24 $$ \mathrm{Biosensor}\ \mathrm{LPG}:0.00270+0.00270+0.00376+0.0288+0.00149+0.0256+0.0811+0.1139+0.0041+0.00544+0.0022+0.0477+0.0503+0.0497=0.419; $$

The results of the static AHP revealed a slight preference for the biosensor LPG, i.e., the one with the fibre along the surface.

Once the global weights of the sub-criteria with respect to each parent policy are obtained, each is incorporated as a variable into the model to which they belong, such as the model of the technical aspects, which includes global sub-criterion weights for technological efficiency, reliability and safety technology. These global weights are then multiplied by the outputs of each model that is local to the weights of the three alternatives in each sub-criterion.

In accordance with the AHP model, first, the target (goal) of the survey is defined, i.e., to evaluate a new health technology to assay Tg in patients with differentiated thyroid cancer. Five main dimensions corresponding with the sub-dimensions were identified, which together compose the so-called dominance hierarchy. In particular, the following main dimensions (criteria) were chosen: technical, organizational, economical, clinical, and social, legal, and ethical aspects. Figure 1 (shown above) reports the overall hierarchy of both the criteria and sub-criteria.

Then, a questionnaire composed of two parts was developed. The first part of the questionnaire (Table 3) included 13 items, each semantically connected to dimensions of the quality of a service identified in the dominant hierarchy in Fig. 1. The respondents were asked to express their degree of agreement/disagreement with each statement by choosing one of the five answers provided by the Likert scale: strongly agree, agree, uncertain, disagree, and strongly disagree. The second part of the questionnaire (Table 4) related to the AHP and consisted of 13 comparisons, divided into 5 batteries, each containing some comparisons; the first battery regarded the pairwise comparison between the 5 main dimensions; each of them included a battery of pairwise comparisons between the three corresponding sub-dimensions.

**Table 3:** First part of the questionnaire based on the Likert methodology

Please indicate with an X your level of agreement/disagreement with the following statements					
	strongly agree	agree	uncertain	disagree	strongly disagree
1. The reliability of the medical equipment is satisfactory	5	4	3	2	1
2. The technological efficiency is appropriate for the type of treatment	5	4	3	2	1
3. The technological safety is high to reduce any danger	5	4	3	2	1
4. The procedural complexity is quite easy and well organized	1	2	3	4	5
5. The preparation of the medical staff is adequate	5	4	3	2	1
6. The staff is adequately available	5	4	3	2	1
7. The possible side effects of the treatment are not very dangerous for the patients	5	4	3	2	1
8. The clinical efficacy of the treatment is evaluated as good	1	2	3	4	5
9. The cost of the treatment is appropriate for the quality offered	5	4	3	2	1
10. The duration of the treatment is not long in relation to the type of disease	5	4	3	2	1
11. In the hospital, there is a good respect for social principles	5	4	3	2	1
12. The legal issues are respected	1	2	3	4	5
13. The ethical principles are respected	1	2	3	4	5

**Table 4:** Second part of the questionnaire based on the Likert methodology

	(pairwise comparison between sub-dimensions used to evaluate the quality of the structure)									
	Extremely less important	Much less important	Less important	Slightly less important	Equally Important	Slightly more important	More important	Much more important	Extremely more important	
Technical Aspect										
Reliability										Technological Efficiency
Reliability										Technological Safety
Technological Safety										Technological Efficiency
Clinical Aspects										
Clinical Efficiency										Effectiveness
Clinical Efficiency										Side Effects
Effectiveness										Side Effects
Organizational Aspect										
Procedural Complexity										Human Resources
Economic Aspect										
Investments										Usefulness
Investments										ROS
ROS										Usefulness
Social, Legal, Ethical Aspects										
Respect for social aspects										Respect for ethical principles
Respect for social aspects										Respect for legal issues
Respect for ethical principles										Respect for legal issues

For each comparison, the respondents were asked which of the two parameters, in his own experience, is more important in determining the quality of the service, according to the Saaty’s scale [9]. The questionnaire was administered to a group of 80 experts in technologies for oncological treatment care at the National Hospital A.O.R.N “A. Cardarelli” of Naples.

### Processing the questionnaire data based on the Likert scale

**Fig. 4:**
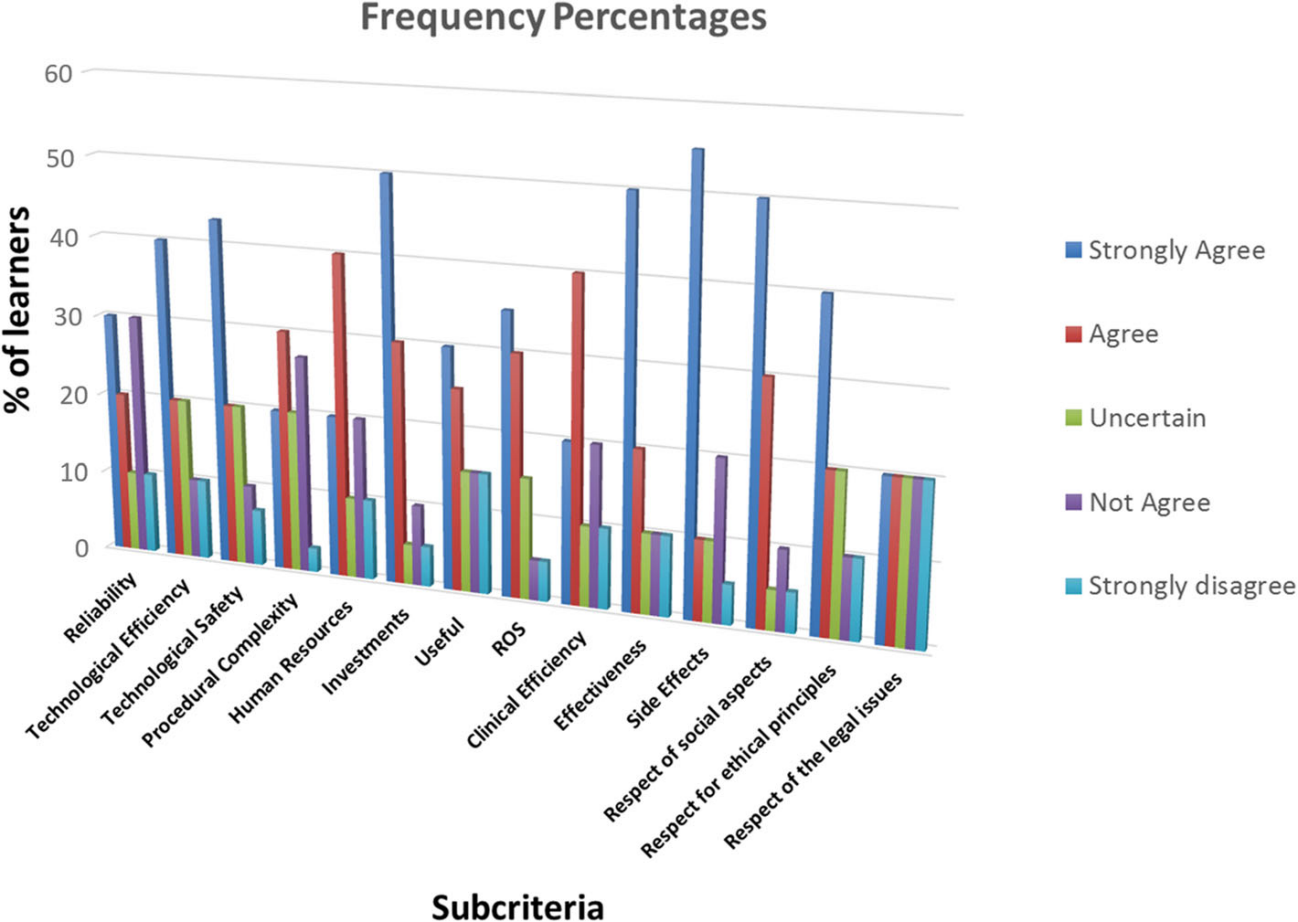
Graph of processed Likert (Subcategories) data

The first step was to evaluate the percentage of experts who provided a specific response based on their satisfaction with the quality of service provided. The frequency percentages of the responses are reported in Fig. 4. The obtained data revealed that the experts expressed relatively high satisfaction percentages (rates) for each item, and in particular those items relating to the effectiveness, the side effects and the respect for social aspects.

### Calculation of local and overall priorities

The eigenvalue method, as proposed by Saaty [9], was used to evaluate the local priorities. In particular, the Global weights of the sub-criteria with respect to their parent criterion are calculated through the AHP by building the matrix of pairwise comparison (Table 2) and then calculating the maximum eigenvalue of the matrix and of the related eigenvector which, once normalized, will represent the vector of the priority weights of the element of the hierarchy that is being considered.

Therefore, for the main categories, we have the following order of alternative variables:

- Technical Aspects weight: 0.0404
- Organizational Aspects weight: 0.08065
- Clinical Aspects weight: 0.2413
- Economic Aspects weight: 0.4933
- Social, Legal, and Ethical Aspects weight: 0.1439

The next step was to calculate the overall weight of each sub-criterion by multiplying the sub-criterion priorities by the priority of their parent criterion [65]. For example, the weight of the sub-criterion “Technical Aspects” is 0.0404.

The matrix and weight analysis of the main categories and sub-categories are shown in Table 5.

**Table 5:** Analysis of the matrix and weights of the main categories and sub-categories

	Reliability	Technological Efficiency	Technological Safety	Weight	Overall weight
Technical Aspects					
Reliability	1	1	3	0,4286	0.0173
Technological Efficiency	1	1	3	0,4286	0.0173
Technological Safety	1/3	1/3	1	0,1429	0.0058
	Reliability	Technological Efficiency	Technological Safety	Weight	Overall weight
Clinical Aspects					
Clinical Efficiency	1	1	3	0.4286	0.1034
Effectiveness	1	1	3	0.4286	0.1034
Side Effects	1/3	1/3	1	0.1429	0.0345
	Procedural Complexity	Human Resources	-	Weight	Overall weight
Organizational Aspects					
Procedural Complexity	1	5	-	0.8333	0.0672
Human Resources	1/5	1	-	0.1667	0.01345
	Investments	Usefulness	ROS	Weight	Overall weight
Economic Aspects					
Investments	1	1/3	1/5	0.1140	0.0563
Usefulness	3	1	1	0.4054	0.200
ROS	5	1	1	0.4806	0.237
	Respect for social aspect	Respect for legal aspect	Respect for Ethical aspect	Weight	Overall weight
Social, Legal, Ethical Aspects					
Respect for social aspect	1	3	1/5	0.1884	0.0272
Respect for legal aspect	1/3	1	1/7	0.0810	0.0117
Respect for Ethical aspect	5	7	1	0.7306	0.105

**Fig. 5:**
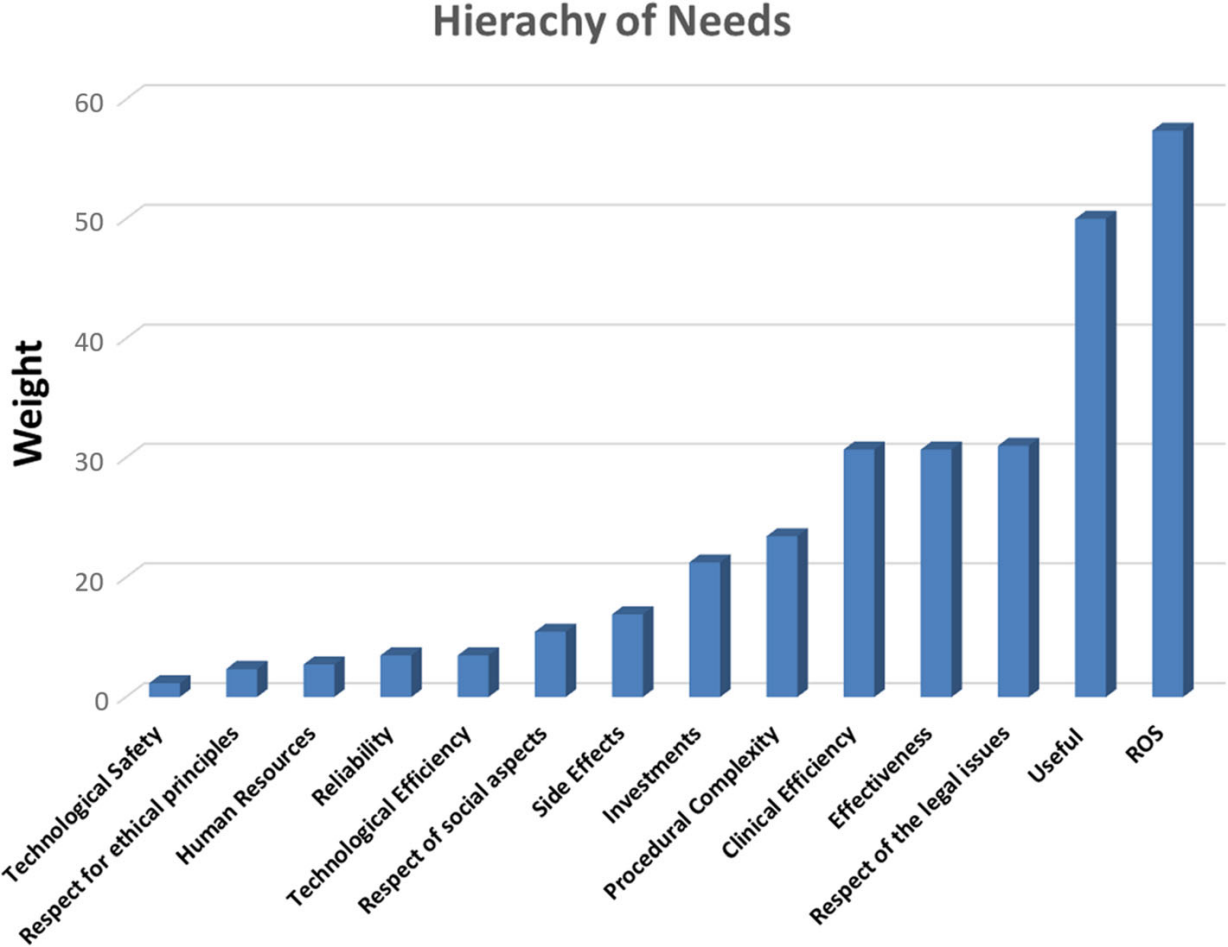
Graph of processed (AHP hierarchy of needs) data

From these calculated values, which represent the absolute weights of each sub-criterion, the “hierarchy of needs” of the users could be defined (Fig. 5), the top priority could be deduced from the histogram. Particularly high importance was attributed to ROS, usefulness, respect for legal issues, effectiveness and clinical efficiency. In contrast, little importance was attributed to the technological safety, the respect for ethical principles, human resources and reliability.

### Comparison and aggregation of the obtained data

The next step was to compare the obtained results and to combine the information produced these two techniques to create a practical tool that can facilitate the decision-making process, thus improve its perceived quality. The parameters that users evaluated as unsatisfactory are characterized by a low value from the Likert method and high values from the AHP.

The hierarchy of needs reveals that technological safety, human resources, and other parameters, such as side effects, usefulness and the respect for social issues, were highly valued with regard to customer satisfaction. Therefore, the experts’ perceptions regarding quality were higher when the degree of user satisfaction was evaluated than when the same item was evaluated by the Likert scale.

Low satisfaction with the clinical efficiency, the effectiveness, the usefulness and the ROS was observed. Therefore, satisfaction with these parameters must be improved to increase the quality of service.

This same reasoning can be extended to all the items, so users’ needs can be understood by applying the AHP, and the users’ satisfaction can be understood by applying the Likert method. In fact, the AHP results confirm a slight preference for the LPG biosensor.

## Conclusions

A new health technology for the assay of Tg in patients with differentiated thyroid cancer was evaluated through two methodologies: the Likert scale and the AHP, to improve its service from an organizational point of view. The evaluation of a health technology is a clear example of a multicriteria decision problem that has a high multitude of interconnected variables. In particular, this choice involves a particularly serious application, and it requires evaluating either a traditional procedure such as cytological analysis or one of two optoelectronic systems, which have completely different characteristics and organization from the first.

In conclusion, this method can be used to increase the quality of a service while avoiding wasting time and costs. Instead, these resources can be invested in improving those service criteria for which users’ satisfaction is low, as revealed with the Likert method, and those that the users assign high importance, as revealed by the AHP.

## Data Availability

The datasets used and/or analysed during the current study are available from the corresponding author on reasonable request.

## Abbreviations

AHP: Analytic Hierarchy Process
CI: Consistency index
CR: Consistency ratio
RI: Random consistency index
